# 

**Published:** 1903-10-17

**Authors:** 


					1HE HOSPITAL. "The Standard of highest purity."?Lancet. October 17th, 1903,
CADBURY'S W CADBURY'S
COCOA (t, m-m. i COCOA
A PERFECT FOOD. nq druqs qr alkalie8 U8BD<
vsaorr Western Infirmary
No. 890. /Vol. XXXV. [aNew^fp^] Saturday,
Oct. 17th, 1903.
pmbBoriptionT^ms,] pR|CE THREEPENCE. fJ

				

